# Study on flow distribution pattern and conductivity of porous media in bioretention cells

**DOI:** 10.1080/21655979.2021.1997131

**Published:** 2021-12-22

**Authors:** Yajun Wang, Yunmei Si, Sheng Yang, Rajendra Prasad Singh

**Affiliations:** a School of Civil Engineering, Lanzhou University of Technology, Lanzhou, China; b Western Engineering Research Center of Disaster Mitigation in Civil Engineering of Education, Lanzhou University of Technology, Lanzhou, China; c School of Civil Engineering, Southeast University, Nanjing, China

**Keywords:** Bioretention Cell, Flow distribution, Microbial behavior, Conductivity, Numerical simulation, Tracer test

## Abstract

To evaluate the long-term performance of bioretention cell (BRC), a study was undertaken to assess the flow distribution and conductivity. Despite initial conductivity of the original medium being the common predictor of hydraulic performance, most of the BRCs are affected by conductivity variations during actual operation. This happen due to the fact that microbial behavior plays an important role in the conductivity variations. This linkage may occur when bacteria as inert colloids transports between particles and biodegrades dissolved pollutants, either promoting or retarding flow distribution and conductivity in BRC. Flow distribution was determined by numerical simulation and tracer test, and the correlation between conductivity and flow distribution was revealed by conductivity experiment coupled with flow distribution analysis. Results revealed a non-uniform flow distribution in BRC, and seepage flow in submerged zone was virtually impossible push flow. Conductivity had an inversely proportional relationship with hydraulic efficiency where hydraulic efficiency reached the highest value (0.297) under a low hydraulic conductivity (0.000107 m/s, approximately *K*/*K*_ini_ = 0.79). Primary cause of hydraulic capacity reduction was the initial permeability decrease due to medium structure changes. Results revealed a sharp upward trend followed by a slight decrease, and then, stabilized to a stable infiltration stage. Permeation process of sewage influent was similar to the one of potable water where the permeability reduced to 0.000102 m/s after 450 h and declined continuously. Thus, it is clear that flow distribution and conductivity in bioretention must be estimated more accurately on a microscopic scale.

## 1. Introduction

Hydraulic conductivity (*K*) is the most important parameter to evaluate operating performance and lifespan of bioretention cell (BRC). The design and maintenance requirements of BRC are directly affected by *K* value; moreover, *K* also affects the decontamination capability of BRC. For example, the down flow bioretention (DFBRC) with a lower *K* can easily lead to more frequent overflows, therefore, decreasing the annual mass of pollutants captured by the system. Microbial community plays an important role in the ecological treatment system [1]; meanwhile, analysis of the experimental data reveals a clear relationship between the spatial structure of particles and the ability of biomass to reduce its hydraulic conductivity [2]. The microbial clogging of ecological treatment system [constructed wetland and BRC] has been an object of intense study in the last few decades. Bergman *et al*. (2011) [3] suggested a final *K* between 30% and 70% of the initial values for the infiltration trenches after 2 years and 9 months of operation. Consequently, it is critical that these potential decreases in *K* are taken into account when designing systems.

At present, the design of BRC is commonly based on the initial hydraulic conductivity [*K*_ini_]. Most of the researchers in system design has confined to the macroscopic *K*_ini_, which is still a research method of ‘black box’. Costumer *et al*. (2009) [4], undertook a review of 37 biofilters in Australia and found that a large number had measured hydraulic conductivity of around 25–50% of their initial values. According to Wilson *et al*. (2015) [5], *K*_ini_ reduction is attributed to soil compaction during basin construction. Therefore, as the most important design parameter, *K*_ini_ is only an initial value based on the packing ratio of the medium, and it could be changed during operation. In addition, even if the media used in the DFBRCs have the similar *K*_ini_, but results still revealed different trend for the permeation performance due to the difference in the flow disorder during operation; the particles compaction degree will be different under various influent loads, which result in the system that cannot achieve the desired efficacy. Therefore, the primary focus of these studies was the macro-performance rather than the process identification. To open ‘black box’, its indispensable approach from the micro scale, the flow distribution must be simulated and analyzed on a porous medium, The formation, decomposition, and exploitation of hydrates are constrained by the state of aqueous interfaces, and the state and behavior of interface water is remarkably different from the well-known behavior of bulk water due to the interface effects [6]. Which considering a spatially varying nature at micro scale, which leads to some inabilities in describing some important interfacial phenomena and properties [7]. Meanwhile, the effect of flow distribution on the conductivity coefficient must be revealed, and thus, the hydraulic conductivity is more accurately estimated during operation a non-uniform flow distribution in BRC and seepage flow in the submerged zone was virtually impossible push flow. The flow distribution simulation in porous mediums in the literature can been seen in Table 1.

**Table 1.** The flow distribution simulation in porous mediums

Application	Material	Simulation	Porous media -simulation	Reference
Fluid saturations	Sand, oil and water	Lattice Boltzmann Method	Input structure and visual observations	[8]
Permeability of porous materials	Bentheimer sandstone,	Lattice Boltzmann Method	Input structure	[9]
Permeability of filter cakes	Glass beads	Lattice Boltzmann Method	Input structure	[10]
Permeability and tortuosity of porous materials	Glass beads	Lattice Boltzmann Method	Input structure	[11]
Permeability, tortuosity, and packing of porous materials	Lava	Random Walk Simulations	Input structure	[12]
Permeability and tortuosity of porous materials	Glass beads	Random Walk Simulations	Transport properties by tracking ion	[13]
Permeability and tortuosity of porous materials	Sandstone	Random Walk Simulations	Comparison with medical CT	[14]
Bedstructure coordination number and packing distribution	Glass beads	Optimization method	Homogeneity and isotropy	[15]

Numerical simulation has become an important tool for the design and operation of water treatment reactor, the conventional design is based on ideal plug flow reactor (PFR) model, ideal completely stirred reactor (CSTR) model and tank in series (TIS) model [16] with significant differences in their performances. For example, the flow characteristics of the constructed wetland are usually simulated using the ideal PFR model and ideal CSTR model; among these, ideal PFR model was recommended by USEPA and WPCF [17,18], to describe the flow regime in wetlands. However, the tracer concentration would emerge a ‘tail’ after reaching its peak in the actual tracer concentration-time curve. Therefore, the ideal PFR CSTR models cannot completely simulate the actual asymmetric tracer concentration-time curve. The degree of actual reactor flow regime deviating from the classical ideal reactor flow regime is very high.

In particular, the biochemical process can be affected by the reactor flow conditions because the biomass, substrates, and inhibiting compounds can be distributed in different reactor zones; such as dissolved oxygen as an inhibiting compound will inhibit the nitrification reaction when the content is low, and many refractory organics often have their own toxicity and inhibit the growth of microorganisms. This implies that the biochemical process can occur with a different kinetic depending on hydrodynamic conditions [19]. The microbial growth is mainly related to the biomass formation in the filler system, and diffusion and convection provide a powerful condition for nutrient transportation for microbial growth, whose availability therefore can also be influenced by the liquid flow. However, biomass detachment means removal of biofilm pieces, which can be carried away by the flow [20]. Inhibition could also happen due to the accumulation of some inhibitory compounds in specific reactor zones including dead angle, short-circuit flow, and cake ditches. Residence Time Distribution (RTD) is a common analysis method for describing the reactor hydraulic behavior and exploring the existing flow deviations from ideal flow [21]. RTD curve can be used to evaluate the infiltration phenomenon and the degree of mixing rate in bioreactors [22].

The hypothesis of this study was the four-stage theory of ‘decrease-increase-then decline-stability’ of BRC seepage coefficient. Therefore, current work was aimed to provide an overall assessment of the flow distribution and hydraulic conductivity behavior of BRCs. The flow distribution was determined by numerical simulation and tracer test, and the correlation between conductivity and flow distribution was revealed by conductivity experiment coupled with flow distribution analysis results. Current study also aimed to investigate the hydraulic conductivity behavior can be judged more accurately on the microscopic scale, which can provide more accurate control parameters for practical applications.

## 2. Materials and methods

### 2.1. Experimental setup

**Figure 1.**
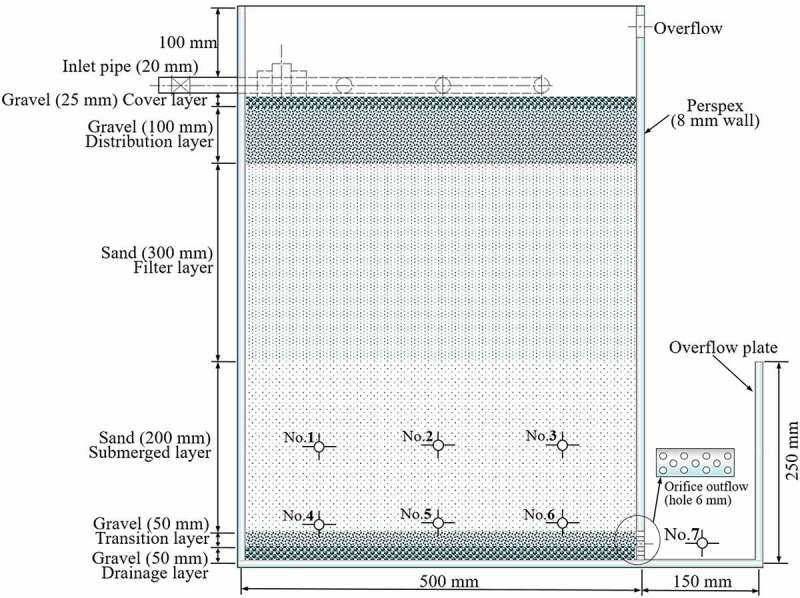
Schematic diagram of experimental unit (seven sampling points: No. 1, No. 2, No. 3, No. 4, No. 5, No. 6, No. 7)

For current study, two DFBRCs were constructed from nonopaque Perspex. The inner dimensions of one reactor were 500 mm (L) × 800 m (H) × 300 mm (W). The inner walls of the reactors were sandblasted to prevent preferential flow along the edge (Figure 1). The inlet PVC tubes (20 mm in diameter) were attached on the top of DFBRC, and the orifice outlets (50 holes, 6 mm) were placed at the bottom of DFBRC and extended into a vertical riser plate. By overflowing the plate, the water level could be kept constant at the bottom of the cell, thereby creating the submerged zone (SZ).

In the filter media, six sampling pipes (No. 1, No. 2, No. 3, No. 4, No. 5, and No. 6) were installed at 100 and 200 mm from the bottom and 125, 250, and 375 mm from the left wall, and one sampling pipe (No. 7) was installed at 50 mm from the bottom in the effluent trough (Figure 1). These pipes were made of 6 mm rubber tubes traversing the entire cell diameter to ensure that the samples were representative of the entire filter.

The design criteria of DFBRCs is according to the Australian FAWB adoption guidelines [23]: a top gravel cover layer (125 mm) to prevent the washout of the overlying soil, a filter layer of washed sand (300 mm, diameter 0.15–1.00 mm), a sand submerged layer (200 mm, diameter 0.25–0.50 mm), following transition layer of coarse sand (50 mm, diameter 0.50–1.00 mm) and the bottom gravel drainage layer (50 mm, diameter 3–5 mm) with an orifice outlet, which was connected to the effluent trough. Consequently, DFBRC had an initial hydraulic conductivity of around 0.135 mm/s with 0.1387 porosity. The reactors were kept without any vegetation in order to assess the influence of compaction (due to hydraulic loading) on hydraulic conductivity. Take great care to waterproof seals at connection points. Use collars on outlet pipes at the point where it traverses the wall. This can be tricky, especially to achieve compaction around the seal. Alternatively it is feasible to use shotcrete to create a large collar extended across the basin surface. A filter fabric can be used around the top of inlet pits and underneath inlets and sediment forebays to prevent preferential flows underneath and down the sides, where the structures are embedded below the filter media surface [23].

### 2.2. Experimental procedure

Two lab-scale reactors were placed in our laboratory located in Nanjing, China, to ensure that the only inflow water received was by controlled dosing, and these were then flushed 3 times during 3 days with deionized water, to promote natural biofilm development. The design of the experiment was aimed at replicating the real time conditions as much as possible while ensuring the degree of control and repeatability necessary to elucidate the impacts of the experimental parameters.

Lab-scale experiments were conducted over 30 days (from July 1^st^, 2016, under summer conditions) in triplicate; two reactor tests (‘Test A’ and ‘Test B’) were designed to emulate the flow distribution and measure the hydraulic conductivity. Test A was performed by tracking the flushing of deionized water dosed with sodium chloride tracers for describing the flow distribution under four hydraulic loading rates (0.5, 1.0, 1.5, and 2.0 m^3^/m^2^·d). Contrast to Test A, Test B was continually dosed with synthetic sewage and Test B was packed and manually compressed to the same bulk density as that in Test A. To ensure consistency in the inflow and avoid logistical issues in collecting large swage Test B, synthetic sewage was used during the entire Test B experiment with C/N = 2:1. Synthetic sewage was made from glucose, NH_4_Cl, K_2_HPO_4_, NaHCO_3_, FeCl_2_ · 4H_2_O, humic acid and trace elements to simulate real domestic wastewater, as follows: chemical oxygen demand (COD): 150 mg/L; total suspended solids (TSS): 40 mg/L; total nitrogen (TN): 64 mg/L; total phosphorus (TP): 4 mg/L; Sodium Bicarbonate (NaHCO_3_): 90 mg/L; iron (Fe): 10 mg/L; cobalt (Co): 1 mg/L and nickel (Ni): 0.5 mg/L. Subsequently, to measure the change in *K* with time under 24 different hydraulic loading rates (HLR), from 0.4 m^3^/m^2^·d in Test B, the HLR was increased by 0.1 m^3^/m^2^·d at intervals of 48 h until HLR reached 2.5 m^3^/m^2^·d, immediately, the HLR was raised to 3.0 m^3^/m^2^·d after 48 h, and then, the HLR was boosted to 3.5 m^3^/m^2^·d after 48 h. The annual mass of pollutants captured by the system was determined by the system treatment capacity, which is the joint action of filler filtration, adsorption and biological reaction under the corresponding HLR, which is subject to the pollutants migration and transformation mechanism in porous media.

### 2.3. Flow distribution analysis method

#### 2.3.1. Mathematical model

A comprehensive, two-dimensional model was developed for DFBR (Figure 1) and considered using a commercial code Fluent 6.22 (Fluent Inc., USA). Porous media were modeled by adding a momentum source term (*S_i_*) to the standard fluid flow equations (De boer and Didwania, 2004) [24]. Thus, the momentum balance in the porous media could be defined as Equation (1).

(1) $$\rho v\cdot \nabla v=-\nabla p+\nabla \cdot \left[\mu \left(\nabla v+{\left(\nabla v\right)}^{T}\right)\right]+S_{i}$$

where *ρ* is the density of the liquid, *v* is the vector velocity of the liquid (m/s), p is the static pressure (Pa), and *μ* is the viscosity (Pa·s). *S_i_* was composed of two parts: a viscous loss term (the first term on the right-hand side of Equation (2)) and an inertial loss term (second term on the right side of Equation (2)):

(2) $$S_{i}=-\left(\overset{3}{\underset{j=1}{\sum }}D_{ij}\mu v_{j}+\overset{3}{\underset{j=1}{\sum }}C_{ij}\frac{1}{2}\rho v_{mag}v_{j}\right)$$

where *S_i_* is the source term for the *i*^th^ (*x, y*, or z) momentum equation, and *D* and *C* are the prescribed matrices. In case of simple homogeneous porous media:

(3) $$S_{i}=-\left(\overset{3}{\underset{j=1}{\sum }}\frac{\mu }{\alpha }v_{i}+\overset{3}{\underset{j=1}{\sum }}C_{2}\frac{1}{2}\rho v_{mag}v_{i}\right)$$

where 1/α is the viscous loss coefficient (m^−2^) and *C*_2_ is the inertial loss coefficient (m^−1^).

In case where modeling a laminar flow through the porous media in this study which was similar to a packed bed, the viscous loss and inertial loss coefficients in each component direction could be identified as (De boer and Didwania, 2004) [24]:

(4) $$1\;/\;\alpha =\frac{150}{d_{p}^{2}}\frac{{\left(1-\varepsilon \right)}^{2}}{\varepsilon^{3}}$$

(5) $$C_{2}=\frac{3.5}{d_{p}}\frac{\left(1-\varepsilon \right)}{\varepsilon^{3}}$$

where *d_p_* is the mean particle diameter (mm), and *ε* is the void fraction.

The computational grid files of the two-dimensional model were generated by Gambit 2.2.30 [24,25]. The velocity inlet was adopted as inlet boundary conditions, and the pressure outlet was adopted as outlet boundary conditions. Interior boundary conditions were used for the filter layer, and fluid boundary conditions were used for the internal unit area, whereas the surface wall was adopted as the surrounding boundary conditions. The simulation conditions and the geometric parameters of the model are presented in Figure 1 and Table 2.

**Table 2.** Geometric parameters and simulation parameters of the layered bioretention model

Parameters	Cover layer	Distribution layer	Filter layer	Submerged layer	Transition layer	Drainage layer
Height (mm)	25	100	300	200	25	25
Mean diameter (*d*_1_, mm)	12	6	2	2	6	15
Void fraction (*ε*_1_, %)	44.5	44	30	33	37	45
Viscous resistance coefficient (1/α, m^−2^)	3.641 × 10^6^	1.534 × 10^7^	6.806 × 10^8^	4.684 × 10^8^	3.265 × 10^7^	2.213 × 10^6^
Inertial resistance coefficient (*C*_2_, m^−1^)	1.837 × 10^3^	3.834 × 10^3^	4.537 × 10^4^	3.263 × 10^4^	2.684 × 10^3^	21.408 × 10^3^

#### 2.3.2 Tracer test method

Tracer tests were conducted using sodium chloride (NaCl). The density of NaCl solution was far greater than deionized water. The effluent electrical conductivity (EC) was tested and recorded for RTD calculation. 300 mL of NaCl solution (250 mg/L and 1000 mg/L, respectively) was added into volumetric flasks with different kinds of sand and gravel. Electrical conductivity had no significant change both within 12 and 24 hours and the concentration of NaCl was linear with EC (R^2^ = 0.99878). For each trial, the box was first saturated by deionized water piped from a water storage tank. Background deionized water’s EC was recorded for each trial. Tracer tests were started with a fast pulsing injection of NaCl solution (100 mL). At the interval of 10 minutes, 50 mL of sample was collected from the effluent. EC of each sample was tested by a conductivity analyzer (DDS-307A, Shanghai Precision Instrument Co., Ltd., Shanghai, China). Calibration was made by testing the reference EC at a known NaCl tracer concentration and water temperature. Linear regression was made to guarantee that more than 90% of tracer concentration was captured for RTD curve in each trial. It was measured with NaCl at the interval of 10 minutes, until the conductivity was not displayed. The total amount of collected effluent was the total amount of captured pollutants and obtained the total amount of captured pollutants.

Earlier studies noted that based on the fluid reactor theory, the concentration measured in pulse tracer experiments was equal to the hydraulic residence time distribution density [26]. The measured conductivity was normalized according to Equation (6):

(6) $$N\left(t\right)=\frac{\left(E\left(t\right)-E_{w}\right)M_{NaCl}Q}{\left(\lambda_{N_{a}}+\lambda_{Cl}\right)M}$$

where *t* is the tracer injection time (h), *N*(t) is the normalized hydraulic residence time distribution density (h^−1^), *E*(t) is the effluent electrical conductivity (s/m), *E*_w_ is the background water conductivity (s/m), *Q* is the inflow rate (m^3^/h), *M*_NaCl_ is the molar mass, 58.44 g/mol, λ_Na_ and λ_Cl_ are the conductivities of Na^+^ (5.01 × 10^−3^ (s·m^2^)/mol) and Cl^−^ (7.63 × 10^−3^ (s·m^2^)/mol), respectively, and *M* is the total amount of tracer (g).

The mean residence time (*t*_m_) and the standard deviation for the average time (*σ*) of the liquid in DFBRC were obtained directly from the particle trajectory model. To compare RTD in different constructions or dissimilar conditions, each parameter must be normalized. Normalized retention time (*t*_θ_) and normalized variance (*σ*_θ_^2^), which were derived from *t*_m_ and *σ* to estimate the hydraulic performance, were, respectively, normalized by Equation (7) and Equation (11). However, neither *t*_θ_ nor *σ*_θ_^2^ were adequate to compare the variable designs. Then, the assessment of the effects of short circuit flow and mixing ability on hydraulic performance by hydraulic efficiency [λ) was introduced by Persson et al. (1999) [27] as shown in Equation (12). Here, λ is the ratio of the peak hydraulic retention time (*t*_p_) and the nominal residence time (*t*_n_). The hydraulic efficiency of DFBRC was high in the higher λ values.

(7) $$t_{\theta }=\frac{t_{m}}{t_{n}}$$

(8) $$t_{m}=\overset{\infty }{\underset{0}{\int }}tN\left(t\right)dt/\overset{\infty }{\underset{0}{\int }}N\left(t\right)dt$$

(9) $$t_{n}=\frac{V}{Q}$$

(10) $$\sigma^{2}=\overset{\infty }{\underset{0}{\int }}{\left(t-t_{m}\right)}^{2}N\left(t\right)dt/\overset{\infty }{\underset{0}{\int }}N\left(t\right)dt$$

(11) $$\sigma_{\theta }^{2}=\frac{\sigma^{2}}{t_{n}^{2}}$$

(12) $$\lambda =\frac{t_{p}}{t_{n}}=t_{\theta }\left(1-\sigma_{\theta }^{2}\right)$$

where *t*_θ_ is the normalized retention time, *t*_m_ is the mean residence time (h) and defined as the gravity position of the residence time distribution curve, *t*_n_ is the nominal residence time (h) and defined as the ratio of pore volume and inflow rate, *σ*^2^ is the variance of the RTD (h^2^), which represented the dispersion range of the tracer concentration curve relative to the distribution mean, *t*_p_ is the peak hydraulic retention time (h) and defined as the effluent time of the tracer maximum concentration, and *σ*_θ_^2^ is the normalized variance that represented the hydraulic distribution divergence.

### 2.4. Hydraulic conductivity

To measure the change in hydraulic conductivity over the experimental time, conductivity measurements were conducted in one month interval. The hydraulic conductivity of each reactor was calculated by measuring the outflow volume per unit time using the standard constant head method (ASTM International D2434-68, 2006) as per Darcy’s Law.

## 3. Results and discussion

The hypothesis of this study was the four-stage theory of ‘decrease-increase-then decline-stability’ of BRC seepage coefficient. To evaluate the long-term performance of bioretention cell (BRC), a study was undertaken to assess the flow distribution and conductivity.

### 3.1. Effect of hydraulic loading rate on flow distribution

**Figure 2.**
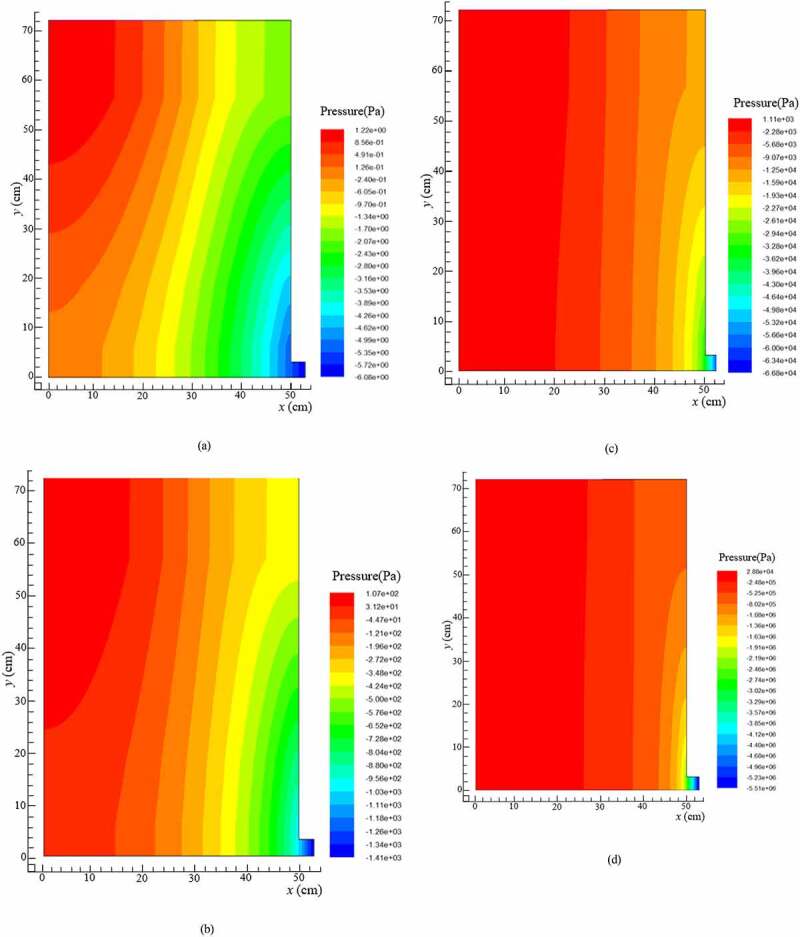
Static pressure in BRC under: (a) 0.5 m^3^/m^2^·d of hydraulic loading rate, (b) 1.0 m^3^/m^2^·d of hydraulic loading rate, (c) 1.5 m^3^/m^2^·d of hydraulic loading rate, and (d) 2.0 m^3^/m^2^·d of hydraulic loading rate

**Figure 3.**
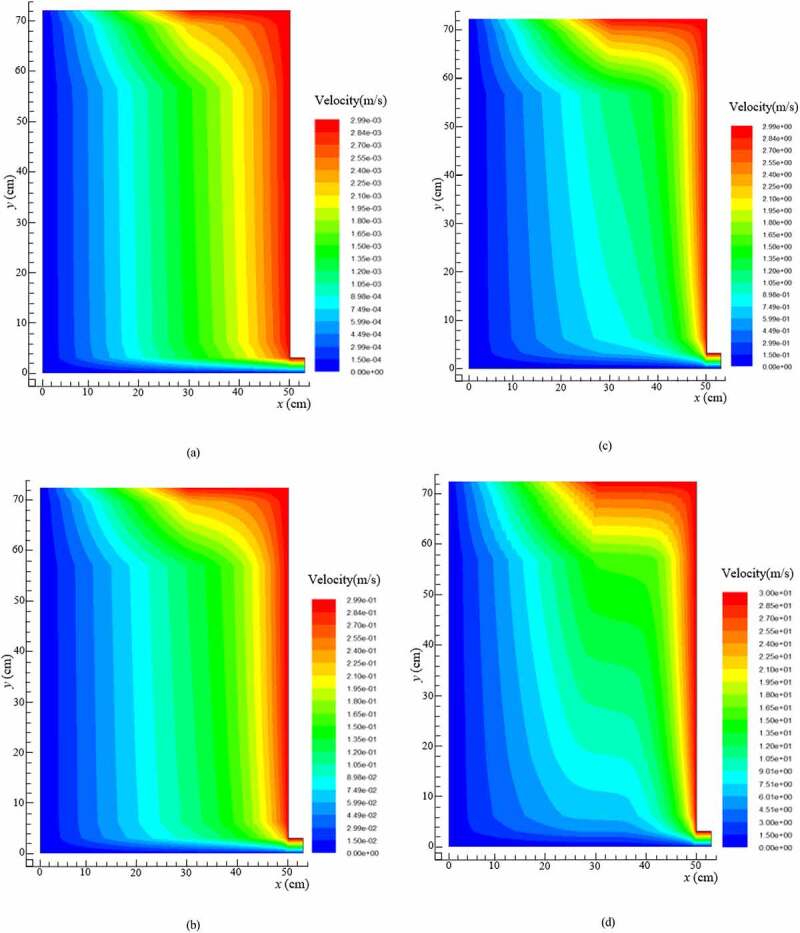
Velocity vectors in BRC under: (a) 0.5 m^3^/m^2^·d of hydraulic loading rate, (b) 1.0 m^3^/m^2^·d of hydraulic loading rate, (c) 1.5 m^3^/m^2^·d of hydraulic loading rate, and (d) 2.0 m^3^/m^2^·d of hydraulic loading rate

The static pressure and the stream function under different hydraulic loading rates were obtained through fluent simulation. As shown in Figure 2, the pressure gradient distribution with the increase of the hydraulic load was gradually from the left to the right side of the reactor. The pressure was mainly distributed in the remaining area of the reactor, which led to an earlier entry into the stable structure at the same time along with the narrower water channel and the larger head loss. The significant reduction in the flow capacity at local or even full regions could be confirmed from the velocity vectors in Figure 3. The results revealed that the flow was smaller and the degree of mixing was weaker. The weak mixing degree not only affected the seepage but also the pollutant reduction.

### 3.2. Comparative analysis of tracer test and simulation results

The hydraulic loading had a significant impact on the short-circuit flow and dead angle in the vertical flow filtration system. The relationship between the hydraulic load and the hydraulic efficiency had a very important role in understanding pollutant removal [28]. Padilla *et al*. (1999) and Nützmann *et al*. [29,30] assumed that there was a positive relation between the degree of saturation and the degree of diffusion, and SZ could improve the flow uniformity. However, both studie [29,30] demonstrated that there was an inverse proportional relationship between the degree of saturation and the degree of diffusion. For this purpose, seven sampling points (Figure 1) in SZ were investigated, and the RTD variety at different hydraulic loading rates and the sampling points at the same hydraulic loading rates were analyzed, respectively. According to Equation (6), effluent electrical conductivity was normalized under different hydraulic loading rates. The RTD distribution curves of seven sampling ports at different hydraulic loading rates were obtained, as shown in Figure 4.

**Figure 4.**
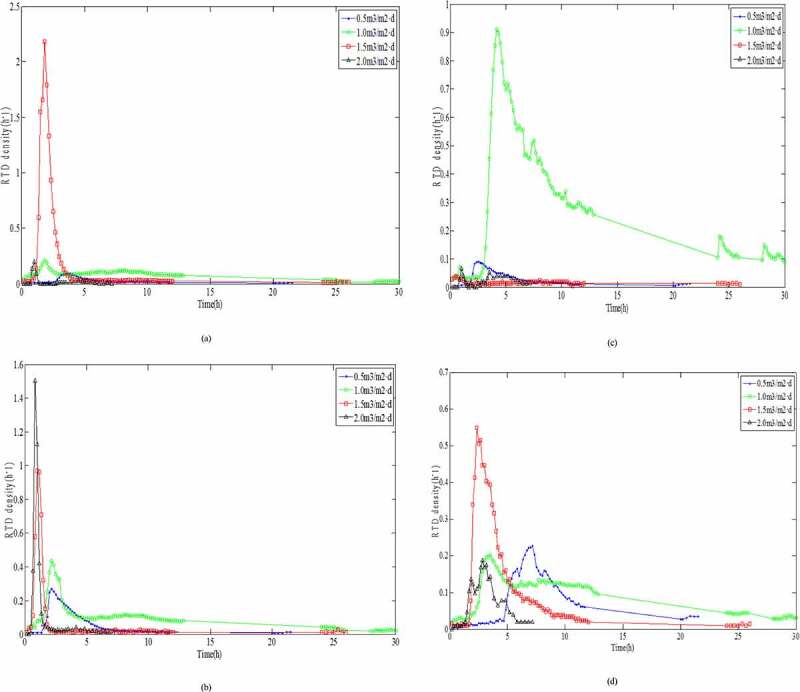
Retention time density distribution curves at different hydraulic loading rates for: (a) sampling point No. 1, (b) sampling point No. 2, (c) sampling point No. 3, (d) sampling point No. 4, (e) sampling point No. 5, (f) sampling point No. 6, and (g) sampling point No. 7

**Figure 4.**
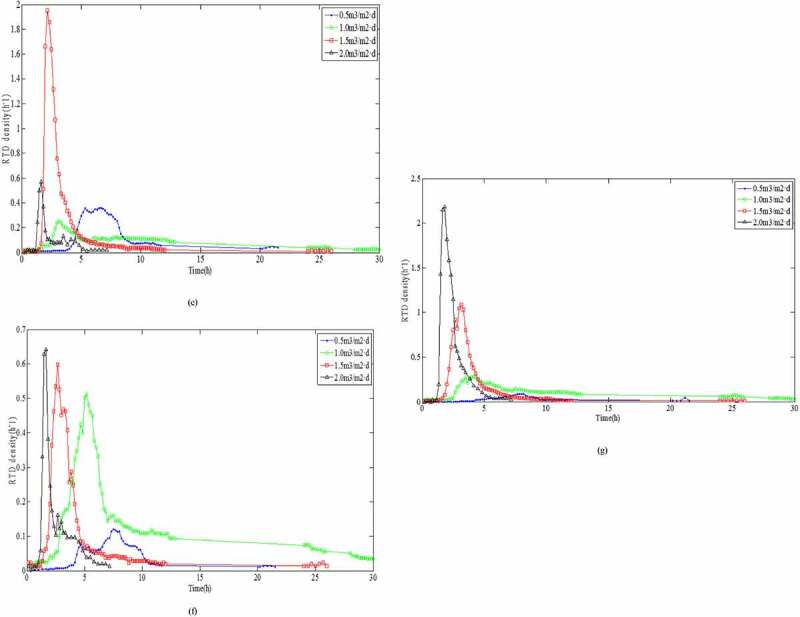
Continued

The results for the variations in the peak appearance times, peak values, peak widths, and tailing length were compared directly as they appeared. According to Darcy Law [31] and Forchheimer Law [32] for the saturated porous media, *K* would be accelerated under higher HLR, where the HRT also decreased. Therefore, it was clear from the RTD curve that with higher HLR values, the peak widths were very narrow, and the higher the peak values appeared at the early period. The results presented in Figure 4(b-e), f, and g showed that the sampling data were all in accordance with the above rules. However, the peak values of Figure 4(a,b) appeared at the lower HLR, because the sampling point No. 1 and No. 4 at up and down position relation were located in the lower left position of the reactor, while the outlet of the reactor was in the lower right position. The flow was infiltrating along the water head, which led to the worst degree of mixing in the lower left position of the reactor. This trend was in complete agreement with the numerical simulation of flow velocity (Figure 3). The peak value of No. 3 (Figure 4(c)) appeared at the lower HLR but there was no obvious value even if the HLR increased to 2.0 m^3^/m^2^·d, which indicated that the partial dead angle existed in the No. 3 region (Figure 1). This might have been due to fewer voids by excessive compaction in the filling process of the filter materials. Low porosity led to a sharp reduction in the flow area and to divert the infiltration direction. This trend was consistent with the results of numerical simulation of pressure values (Figure 2).

**Figure 5.**
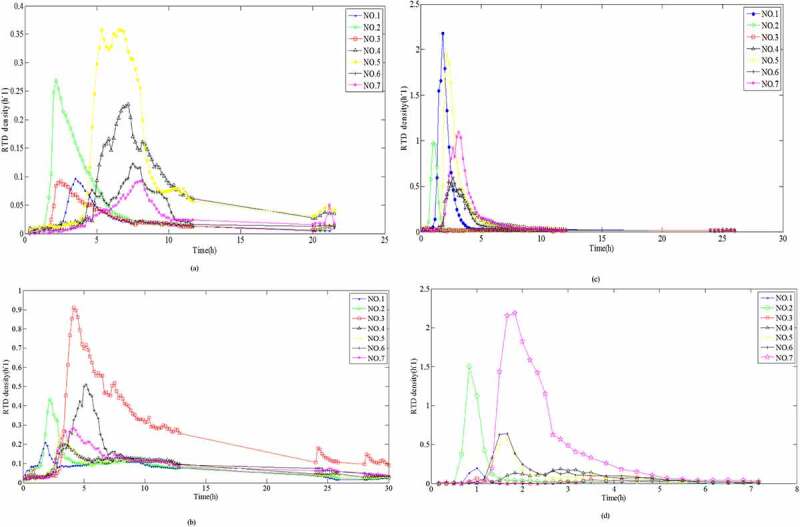
Retention time density distribution curves for different hydraulic loading rates : (a) 0.5 m^3^/m^2^·d; (b) 1.0 m^3^/m^2^·d; (c) 1.5 m^3^/m^2^·d; and (d) 2.0 m^3^/m^2^·d

It was directly compared with the RTD curve variation of each sampling point at the same HLR (Figure 5). The results revealed that the residence time in the curve showed the trend of significant increase and then, decrease as follows: T_1.0_ > T_0.5_ > T_1.5_ > T_2.0_. The hydraulic parameters at different HLR were calculated according to Figure 4 and Equation (7–12). These parameters are presented in Table 2. Under different HLR, both normalized retention time (*t*_θ_) and hydraulic efficiency (λ) showed an increased and then decreased trend: *t*_θ-1.0_ > *t*_θ-1.5_ > *t*_θ-0.5_ > *t*_θ-2.0_ and λ_1.0_ > λ_1.5_ > λ_0.5_ > λ_2.0_. Data presented in Table 2 revealed that *t*_θ_ values were lower than 1 under each hydraulic loading condition, which was an indication of the small ‘dead zone’ in the system. This meant that through molecular diffusion or mechanical dispersion, the tracer entered into the dead zone and it is hard to enter the mainstream channel outflow. It is difficult for the tracer to enter the main infiltrating channel again after flowing into the ‘dead zone’ through molecular diffusion or mechanical dispersion.

During the HLR of 1.0 m^3^/m^2^·d, the effective volume ratio for pollutant removal was the largest and the hydraulic efficiency was the highest. Meanwhile, a larger *σ*_θ_^2^ value illustrated that the flow was more close to completely stirred flow of CSTR model because of the larger distribution divergence. However, as shown in Figure 5 and Table 3, in addition to No. 3, the peak appearance times of the other sampling points: No. 1 ≈ No. 2 > No. 4 ≈ No. 5 ≈ No. 6 > No. 7, showed that the flow was close to the plug flow of the PFR model. According to the reactor theory, in the plug flow reactor, the reaction time for the reactant reducing to the same concentration was shorter; that is, the reaction efficiency was higher [33]. It is also revealed that the seepage flow is virtually impossible push flow of PFR model in SZ of DFBRC.

**Table 3.** Estimated hydraulic parameters based on impulse tracer test data

Hydraulic loading rates (m^3^/m^2^·d)	*t*_m_ (h)	*t*_n_ (h)	*t*_p_ (h)	*σ*^2^ (h^2^)	*σ*_θ_^2^	*N* (h^−1^)	*t*_θ_ (h)	λ
0.5	8.1	33.65	6.9	168.716	0.149	6.750	0.241	0.205
1.0	6.2	16.82	5.0	55.168	0.195	5.167	0.369	0.297
1.5	3.1	11.28	2.8	67.637	0.098	10.333	0.275	0.248
2.0	1.7	8.41	1.6	4.173	0.059	17.000	0.202	0.190

### 3.3 Correlation analysis of flow distribution and hydraulic conductivity

**Figure 6.**
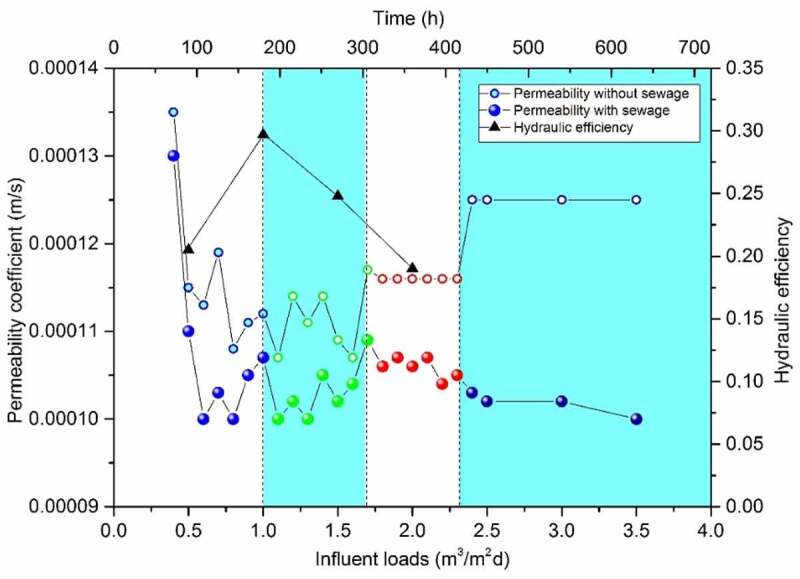
Correlation between permeability, hydraulic efficiency and hydraulic loads

Hydraulic conductivity tests were synchronized in two DFBRC with and without sewage influent under different HLR, which shows in Figure 6. The results revealed a non-linear relationship between *K* and HLR. The reason was the heterogeneous flow distribution increased by the increasing porosity and its non-homogeneous distribution in DFBRC, resulting in an increase of infiltrating flow and exceeding the applicable range of Darcy’s Law. From the mechanics aspect, the study of particle material mechanics found that the particle system had the unique characteristic of the nonlinear response to the external micro action [34].

The results showed that if HLR increased from 0.4 m^3^/m^2^·d to 1.0 m^3^/m^2^·d, the *K* value had the similar downward trend with and without sewage influent when hydraulic efficiency (λ) reached the highest at 1.0 m^3^/m^2^·d. During this period, the microorganism is in the growth stage, and the complete biofilm is not formed on the particles surface, which makes the microorganism have little contribution to the change of flow field [35]. Therefore, the reason for this trend is that when the pressure increases to a certain value, the original structure between the particle swarm begins to deform and the local cavity collapses. Resulted in the formation of smaller voids with increased water head loss increased, and decrease in *K* value. When HLR increased to 1.5 m^3^/m^2^·d, there had been a fluctuating rises due to the dilatancy principle [36], the larger area of the particles was larger than the area of collapse, flow channel became wide, and *K* showed a upward trend.

When HLR increased to 2.5 m^3^/m^2^·d, there were different trends in the two curves, during infiltration of the deionized water, The *K* value decreases slightly and then tends to be stable, which is due to the complete collapse of particles. When the head pressure was insufficient to make the structure deformation, the flow path became more stable and the volumes of the individual bridges approached one another within a characteristic time of about 5 min [37], which ensured the smooth flow. During infiltrating the raw water with sewage influent, *K* decreased gradually, this trend could be explained through the fundamental principles of thermodynamics and biofilm morphology. A closed system tended to become free energy to minimal state, along with the entropy of the system reaching the maximum by thermal diffusion motion, and eventually, forming a uniform distribution equilibrium; therefore, granular structure was ultimately stabilized. At this time, the sewage carrying particulate pollutants and the gradual maturation biofilm had become a restrictive factor to infiltration, resulting in the occurrence of the clogging phenomenon. Formation of thick biofilms may cause pore clogging or the biofilm detachment under the shear force, thus providing a substantial decrease in permeability and a reduction in effluent flow, therefore isolating the pollutant into specific subsurface zones [38]. Thus, understanding biofilm development/detachment and its interaction with fluid flow in porous media becomes important for the successful development and application of many engineering techniques. However, in a porous medium with complex geometry, the local fluid pressure acting normal to the biofilm surface may also play an important role. Local clogging due to biofilm formation may cause an increase in the fluid pressure experienced by the biofilm surface at different locations (Duddu *et al*. 2009) [39]. The results revealed that when *K* was low (0.000107 m/s, *K*/*K*_ini_ = 0.79), the hydraulic efficiency was the highest. This trend ensured the contact and retention times; hence, DFBRC had the ability to remove pollutants efficiently.

The accurate control parameters for practical applications are determined as follows: the BRC should be strictly in accordance with the grading requirements of each layer; the HLR should be controlled at about 1.0 m^3^/m^2^·d, the *K* should be controlled at about 0.000107 m/s and *K*/*K*_ini_ = 0.79.

## 4. Conclusions

Permeability, flow distribution pattern, and hydraulic loading rates and hydraulic conductivity are very important factors in the performance of BRC‒. The flow distribution and the change of microscopic filter structure due to hydraulic loading have an important influence on hydraulic conductivity and thus on the decontamination ability. In this study, the use of a low hydraulic loading rate (approximately, 1.0 m^3^/m^2^·d) could improve the degree of diffusion, resulting in a lower hydraulic conductivity (approximately, 0.000107 m/s, *K*/*K*_ini_ = 0.79), which could achieve the desired permeability and treatment effects. On the basis of CFD simulation and experimental verification, the four-stage theory of ‘decrease-increase-then decline-stability’ of BRC seepage coefficient was put forward. This phenomenon reveals the relaxation process of pressure expansion when the packing particles are disturbed by HLR. The appropriate choice of initial hydraulic conductivity can be a key element in BRC design because it can improve the degree of diffusion and, therefore, indirectly increase the pollutant removal by limiting the volume of water bypassing the system through overflow. Hence, it is clear that the flow distribution and conductivity in BRC must be estimated more accurately on a microscopic scale .
